# The ecological significance of extremely large flocks of birds

**DOI:** 10.1002/ece3.5234

**Published:** 2019-05-06

**Authors:** Anders Pape Møller, Karsten Laursen

**Affiliations:** ^1^ Ecologie Systématique Evolution, Université Paris‐Sud, CNRS, AgroParisTech Université Paris‐Saclay Orsay Cedex France; ^2^ Ministry of Education Key Laboratory for Biodiversity Science and Ecological Engineering, College of Life Sciences Beijing Normal University Beijing China; ^3^ Department of Bioscience Aarhus University Rønde Denmark

**Keywords:** largest colonies, largest flocks, nutrient limitation, population regulation, resource limitation, trophic level

## Abstract

Population size is generally limited by resource availability during and outside the breeding season. Therefore, maximum size of flocks may provide important information on population regulation and the influence of diet and trophic level on maximal degree of sociality. We hypothesized that (a) flock size should increase with nutrient availability; (b) flock size should decrease with latitude because productivity is higher at lower latitude; (c) aquatic habitats should have larger flocks than terrestrial habitats because the former are less accessible; (d) smaller species should have larger flocks because they require overall less food; (e) human‐impacted species that live in perturbed habitats should have smaller flocks than other species; (f) flock size should decrease with increasing trophic level because there is a reduction in biomass due to conversion at each trophic level; and (g) flocks of species depending on ancestral landscapes should have decreased in size in recent years due to human impact (e.g., land‐use). We obtained 1564 observations of flocks that exceeded 100,000 individuals in order to test the predictions listed above. Most effect sizes were small to medium accounting for 1%–9% of the variance, while large effects accounting for 25% or more were only found for total nitrogen used per km^2^ and area used for agriculture. Changes in large bird flocks were caused by habitat degradation and persecution, and temporal decline in size of large flocks revealed changes in nutrient use, reductions in nutrient cycling, and changes in flock size linked to trophic level.

## 1 INTRODUCTION

Population size is commonly limited by resource availability (Newton, 1998). Resource availability limits population size of consumers with the latter matching the distribution of resources in an ideal free fashion (Fretwell & Calver, 1969; Fretwell & Lucas, 1970). Alternatively, consumers may have a despotic distribution with certain individuals monopolizing access to limiting resources (Parker & Sutherland, 1986). While resources may limit the abundance of consumers, it is also possible to argue the other way around by assuming that a larger aggregation of consumers implies a larger amount of resources. Here, we use this latter approach to investigate the factors that contribute to the occurrence of large aggregations of individuals in flocks or colonies.

Large aggregations of birds and other animals require the presence of large amounts of food, implying that such large flocks only occur under the most favorable environmental conditions. In breeding birds, flock size is partly limited by maximum foraging distance (Furness & Birkhead, 1984; Jovani et al., 2015). Therefore, maximum size of flocks may provide important information on population regulation and the influence of ecological factors such as nutrient availability, diet, and trophic level for maximum flock size. This raises the question which are the largest bird flocks and colonies ever observed in the world, and which ecological factors are the main determinants? There are numerous studies of the rarest species in the world, but few or none of the most common species. Here, we provide such a study.

Seabird colonies, and by inference flocks of birds, are important global drivers of the nitrogen and phosphorus cycles (Mosbech et al., 2018; Otero, Peña‐Alberti, Pérez‐Alberti, Osorio Ferreira, & Huerta‐Diaz, 2018). This role for seabird colonies is due to effects of soil, sediment, and water eutrophication.

We hypothesized that (a) nutrient availability sets a limit to flock size through its effects on primary and secondary productivity (Guignard et al., 2017). We suggested that (b) flock size should decrease with latitude (higher productivity at higher latitudes due to permanent daylight in Polar regions during the breeding season and increased loss of ice cover; Meltofte et al., 2013); (c) aquatic habitats should have larger flocks than terrestrial habitats due to higher productivity. Carbon flows are two to three orders of magnitude lower in terrestrial than in aquatic ecosystems (Gounand, Little, Harvey, & Altermatt, 2018), reducing flock size in terrestrial ecosystems; (d) smaller species should have larger flocks due to smaller food demand per individual; (e) human‐impacted species should have smaller flocks than other species due to lower carrying capacity and effects of organic pollutants (Crozier & Gawlik, 2002; Meltofte & Clausen, 2011); (f) flock size should decrease with increasing trophic level due to a reduction in biomass at higher trophic levels (Guignard et al., 2017; Lindeman, 1942); (g) large flocks should decrease in size in recent years due to human impact and exploitation of natural resources (Mokross, Ryder, Côtes, Wolfe, & Stouffer, 2014; Peters, Likare, & Kraemer, 2008); (h) flocks should be larger in more seasonal environments where large flocks can be sustained by seasonal availability of resources (Jakubas, Wojczulanis‐Jakubas, Iliszko, Strøm, & Stempniewicz, 2017); and (i) flock size should increase with increasing amounts of fertilizer for species living in farmland (Pretelli, Baladron, & Cardoni, 2018). We collected a total of 1,564 observations of bird flocks or colonies exceeding 100,000 individuals from 154 species in 69 countries in order to test these predictions.

## 2 MATERIALS AND METHODS

### 2.1 Data collection

**Figure 1:**
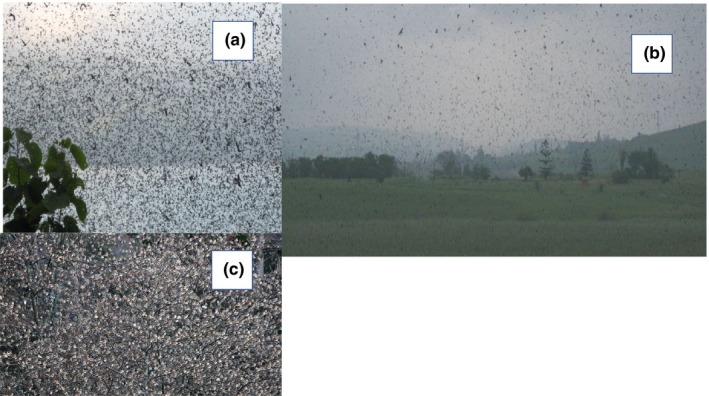
Photographs of large flocks of birds. (a) Amur falcons *Falco amurensis*. Photograph: Péter Féhérvary, (b) barn swallows *Hirundo rustica*. Photograph: Angie Wilken and (c) brambling *Fringilla montifringilla*. Photograph: Tomaž Mihelič

For practical reasons, we used a lower limit of 100,000 individuals to the definition of a large flock, resulting in the accumulation of a total of 1,564 observations of flocks. Three examples of large flocks are shown in Figure 1. If we had relied on flocks that consisted of 1 million or more individuals, this would have resulted in relatively few observations (in this study 329 flocks), while reliance on 10,000 individuals or more across the world would have resulted in so many observations that it would have been unfeasible to obtain even close to full coverage.

This is a study of large flocks and colonies of birds throughout the annual cycle. We used observations of flocks from 1637 to 2019. We defined flocks as aggregations of individuals that remained for shorter or longer time in a location where they consumed food. Therefore, we included flocks of all species that were competing for limited food. However, we did not include migrants that locally may occur in very large numbers for very short periods of time, but do not exploit local food. The classification of species was made according to the variables listed in the remainder of the Materials and Methods.

Flock size was generally derived from visual estimates, counts of a fraction of a flock or counts from photographs followed by extrapolation. Comparison of counts from airplanes and counts from the ground commonly revealed detection rates exceeding 80% for larger species of waterbirds (Broome, 1985; Laursen, Frikke, & Kahlert, 2008; Savard, 1982). If such estimates were reliable, we should expect that repeatability analyses would be reliable and that multiple estimates for a given species would be consistently more similar than a similar number of estimates based on randomly chosen flocks. Flock sizes can also be estimated using estimators based on the number of individuals present in the neighborhood of an average individual (Reiczigel, Lang, Rósza, & Tóthmérész, 2008), but we did not adopt this approach because of lack of data.

We collected data from numerous databases: avif.birds, Czech Republic, Bird Watch Ireland, BirdLife Finland, BTO, United Kingdom, DOF basen, Denmark, artportalen Sweden, artsobservationer, Norway, ChinaBirdReport, China, DDA, Germany, DOPPS, Slovenia, eBird, USA, Naturbasen, Denmark, tiira, Finland, ornitho, Belgium, ornitho, Switzerland, ornitho, Germany, ornitho, France, ornitho, Italy, ornitho, Lithuania, ornitho, Netherlands, Ornithological Societies of North Karelia, South Karelia and Kymenlaakso, Research Gate, Germany, and Svalan, Sweden.

APM asked on Research Gate on 26 May 2016 for information on observations of large flocks of birds exceeding 100,000 individuals, requesting the information to be sent to his email address. This request included a demand for information on species name, date, year, locality, and coordinates. A total of only seven persons responded, and thus, there was only little scope for acquisition of additional information from this general source. The final deadline requested for all entries was 31 March 2017.

We obtained information on 2,271 large flocks. However, 707 of the 2,271 observations were repeats from the same site during the same year. We eliminated such repeats because multiple observations of the same individuals could not be considered statistically independent. Such a large reduction in the number of flocks implies that we must have been close to the number of large flocks that actually exists because we found an increasing number of repeats over time. Elimination of these repeats reduced the number of observations to 1,564 flocks exceeding 100,000 individuals. These 1,564 flocks derived from 154 bird species and 69 countries.

As variables for the statistical analysis, we have used data on fertilizer use per km^2^ and agriculture from EuroStat (2019). Habitat degradation (defined as 0 for pristine or 1 for degraded by humans) and threat status for different species of birds were obtained from BirdLife Datazone (2019). Threat status ranged from +0 to −4, where 0 is least concern, −1 is near threatened, −2 is vulnerable, −3 is endangered, and −4 is extinct. Thus, more negative values imply higher threat status. Bird population size (defined as the size of the global breeding population) and geographic range (defined as the area of the breeding range in km^2^) were from Cramp and Perrins (1977). The distinction between aquatic and terrestrial habitats was based on literature information (Cramp & Perrins, 1977; del Hoyo et al., 1992). That was also the case for aerially foraging bird species that were scored as aerially foraging (1) or not (0) (Cramp and Perrins 1977–1994; del Hoyo et al., 1992). Trophic levels of all species were categorized as 1—primary productivity, 2—consumers, and 3—hyper‐consumers. Finally, body mass was obtained from Dunning (1993), while wing area and aspect ratio were obtained from Vágási et al. (2016) and APM. Summary statistics for the predictor variable and the response variables are reported in Supporting Information Table S1.

### 2.2 Statistical analyses

We tested for consistency by using repeatability (*R*) estimates as indicators of bias (Falconer & Mackay, 1996). We used species as a fixed factor and log_10_‐transformed flock size as a response variable. If a given flock size is a property of each species, we should expect repeatability to be statistically significant. We identified large flocks in 21 species in Europe for the same 21 species in North America. We used a linear regression model to test whether the number of large flocks in Europe was significantly positively related to the number of large flocks for the same species in North America.

We used generalized linear models (GLM) to test for the association between response variables and flock size. Flock size was a normally distributed response variable with an identity link function. We redid all analyses using GLM with species being a predictor variable, other factors being fixed, and other continuous variables being covariates (SAS, 2012). We tested for over‐dispersion of data, but always found adequate support for the models, and there were no cases of statistically significant lack of fit at the level of 0.05.

We used Pearson's product–moment correlation coefficients as estimates of effect size. We calculated effect sizes in terms of Pearson's *r*. Here, we adopted the guidelines of Cohen (1988) as a yardstick, suggesting that *r* = 0.10 explaining 1% of the variance is a small effect, *r* = 0.30 explaining 9% of the variance is an intermediate effect, and *r* = 0.50 explaining 25% of the variance is a large effect. We estimated effect sizes as Pearson's product–moment correlation coefficients, using the equations in Rosenthal (1991), Cooper and Hedges (1994) and Hedges and Olkin (1985).

## 3 RESULTS

### 3.1 Reliability of data

The range in the number of individuals per flock was 100,000–20 billion, mean (*SE*) = 17.59 million individuals (13.06 million individuals).

Some countries had a stunning absence of large flocks. For example, there was only one single observation of a large flock in China despite its very large size and the large number of observers. Locally, there were also some exceptions. For example, Romania, with 505,370 km^2^, did not have a single large flock despite many observers, while France with 551,500 km^2^ had 11 observations of large flocks.

### 3.2 Testing predictions

**Figure 2:**
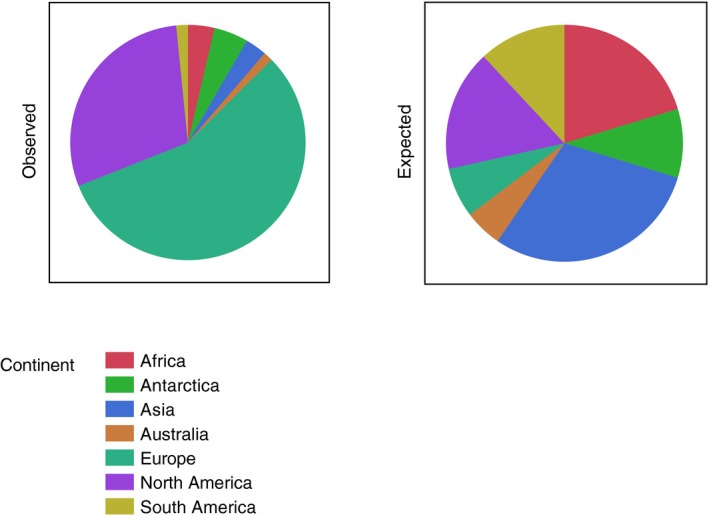
Pie charts of the observed and the expected distribution of large flocks of birds among continents. Expected distribution was calculated according to area

The 1,564 large flocks were unevenly distributed across the seven continents (Figure 2; likelihood ratio *χ* ^2^ (LR) = 3,590.51, *df* = 7, *p* < 0.0001). There were 882 flocks observed in Europe but only 107 expected, while there were 460 observed and 259 expected in North America. There were significantly fewer large flocks in Asia (46 observed) than expected (467 expected). Similarly, there were many fewer in Africa (57 observed flocks) than the expected 318 flocks.

Flock size was repeatable among species as shown by a GLM of log_10_‐transformed number of species being predicted by species identity (LR = 767.635, *df* = 158, 1,405, *p* < 0.0001, repeatability *R* = 0.320; *SE* = 0.014). Likewise, flock size was repeatable among observations within localities and years as shown by an ANOVA of log_10_‐transformed number of species being predicted by species identity, accounting for 73% of the variance in flock size (*F* = 13.780, *df* = 190, 728, *r* ^2^ = 0.33, *p* < 0.0001, repeatability *R* = 0.720; *SE* = 0.016).

There were 21 species with large flocks occurring both in Europe and in North America (log no. flocks of different species in North America and Europe: LR 9.57, *df* = 1, *p* = 0.0066, estimate; *SE* = 0.826 [0.267]). The slope was not significantly different from 1 (*t* = 0.65, *df* = 19, *p* > 0.25). Thus, there were larger flocks of a given species in Europe if there also were larger flocks of the same species in North America.

**Figure 3:**
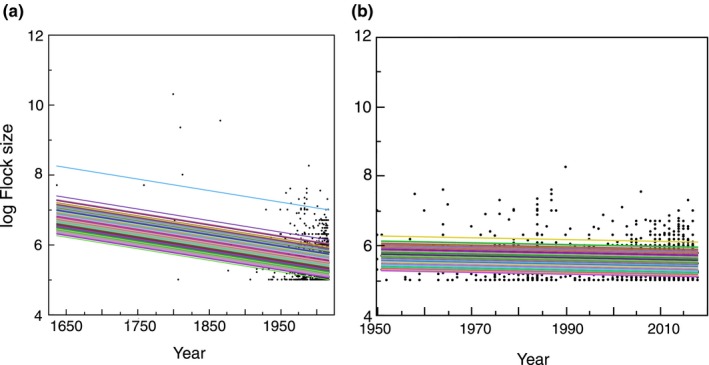
(a) log Number of birds per km^2^ in relation to log total amount of nitrogen per km^2^. The lines show the predicted linear relationships for different species while dots show individual observations. (b) log Flock size in relation to threat status ranging from 0 which is least concern, −1 is near threatened, −2 is vulnerable, −3 is endangered, and −4 is extinct. The lines show predicted linear relationships for different species while dots show individual observations. (c) log Flock size in relation to trophic level. The box plots show median, quartiles, 5 and 95 percentiles, and extreme values. (d) log Flock size in relation to log Population size

**Figure 4:**
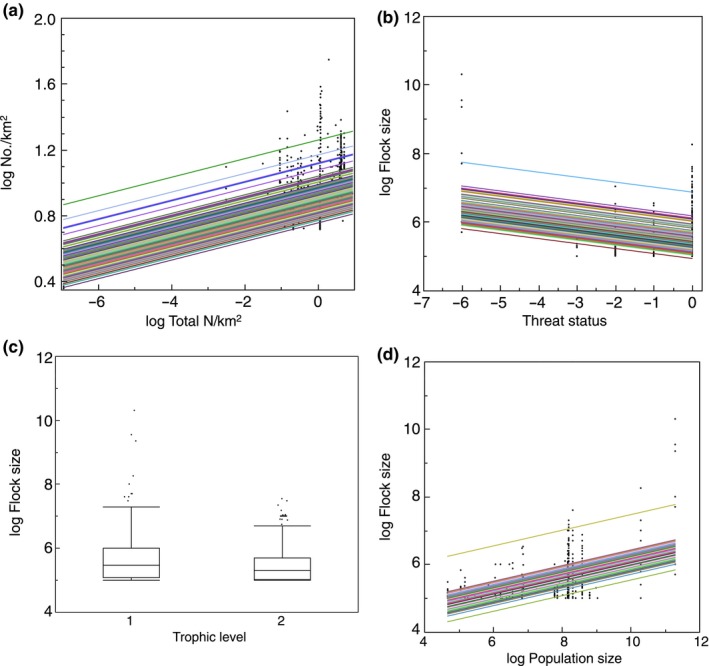
(a) log Flock size of different species of birds in relation to year for 1637–2019 and (b) for 1950–2019. The lines show predicted linear relationships for different species while dots show individual observations

The size of large flocks increased with the level of nitrogen use per square kilometer (Table 1; Figure 3a). There was also a significant positive relationship between the size of large flocks and the area used for agriculture (Table 1; Figure 3b). The size of large flocks decreased with increasing habitat degradation (Table 1). The mean size of large flocks shown in Figure 4a was predicted from the statistical model to decrease from 28 million in 1637 to 0.3 million in 2019 (Table 1; Figure 4a). That was even the case when data were restricted to 1951–2019 (Figure 4b; GLMM with log flock size as the response variable, species as a random effect and year as a covariate: (*F* = 9.35, *df* = 1, 1518, *p* = 0.0023, estimate; *SE* = −0.0026 [0.0008]). Qualitatively, similar results were obtained for 1961–2019, 1971–2019, 1981–2019, and 1991–2019. There was a decrease in flock size with change in threat status implying that flock size was smaller in more threatened species (Table 1). Bird species with larger flocks had larger population size (Table 1). Flock size decreased with latitude with larger flocks at lower latitudes (Table 1). Flocks were smaller in aquatic than in terrestrial habitats (Table 1). Flock size was related to aquatic habitat, latitude, and the interaction between habitat and latitude (LR = 59.35, *df* = 1, *p* < 0.0001) with a negative relationship with latitude for terrestrial habitats (LR = 71.34, *df* = 1, *p* < 0.0001, estimate; *SE* = −0.16 [0.001]), but no relationship for aquatic habitats (LR = 0.05, *df* = 1, *p* = 0.82, estimate; *SE* = 0.0002 [0.0010]). Flock size was larger in aerially foraging species (Table 1). Flock size decreased with increasing trophic level (Table 1; Figure 3c). Flock size was larger during the breeding than during the nonbreeding season (Table 1). Flock size increased with population size (Table 1; Figure 3d). Deviations between flock size and population size may be due to uncertainty in estimates of either variable. Flock size decreased with wing area and increased with aspect ratio (Table 1). Finally, flock size decreased with increasing body mass (Table 1). Most effect sizes were intermediate to small accounting for 1%–9% of the variance with the large effect sizes for total nitrogen use per square kilometer and the area used for agriculture.

**Table 1:** Generalized linear models (GLM) of the relationship between log_10_‐transformed size of large flocks of birds with flock size as the response variable and 15 predictor variables

Variable	LR	*N*	*p*	Estimate	*SE*	Effect size	Estimate [0]	*SE*	Estimate [1]	*SE*
Total *N*/km^2^	308.12	1,457	<0.0001	0.104	0.006	0.46				
Agriculture	845.23	1,457	<0.0001	−0.111	0.003	0.76				
Habitat degradation	34.05	1,564	<0.0001	0.082	0.014	0.15	5.607	0.022	5.444	0.018
Year	89.68	1,564	<0.0001	−0.0053	0.0006	0.24				
Threat status	51.63	1,564	<0.0001	−0.153	0.021	0.18				
Population size	33.54	890	<0.0001	0.158	0.027	0.19				
Range	0.00	890	<0.0001	0.0015	0.0004	0.00				
Latitude	32.09	1,564	<0.0001	−0.0056	0.0010	0.14				
Aquatic habitats	39.82	1,564	<0.0001	0.088	0.014	0.16	5.591	0.020	5.415	0.018
Aerial foraging	14.94	1,564	<0.0001	−0.081	0.021	0.10				
Trophic level	58.24	1,564	<0.0001	0.113	0.015	0.19	5.669	0.032	5.442	0.014
Breeding season	20.51	1,564	<0.0001	−0.075	0.017	0.11	5.479	0.014	5.630	0.037
Wing area	6.26	812	<0.0001	−0.421	0.168	0.09				
Aspect ratio	97.05	816	<0.0001	0.058	0.016	0.34				
Body mass	54.28	1564	<0.0001	0.140	0.019	0.19				

Note

Total *N*/km^2^ is the amount of nitrogen per km^2^. LR is the likelihood ratio statistic, *N* is sample size, and *p* is probability. Estimates and *SE* are reported for the two classes of data whenever predictor variables are dichotomous binomial variables. Effect size is Pearson's product–moment correlation coefficient.

We could not develop a statistical model that included all predictor variables included in Table 1 because that would severely reduce the total number of observations. Therefore, we developed a full model that included nine predictor variables covering the same factors as those reported in Table 1 while maximizing the number of significant predictors (Table 2). Most of these partial effects were similar to those from GLMs with one predictor and one response variable (Tables 1 and 2). Effect sizes were generally small to intermediate explaining 1%–9% of the variance.

**Table 2:** Full generalized linear models (GLM) of log_10_‐transformed size of large flocks are relation to nine predictor variables with only significant predictors included

Variable	LR	*N*	*p*	Estimate	*SE*	Effect size
Total *N* per km^2^	15.08	870	0.0001	−0.056	0.014	0.197
Agriculture	33.85	870	<0.0001	0.478	0.081	0.120
Habitat degradation	5.60	870	0.018	0.056	0.024	0.112
Threat status	23.88	870	<0.0001	−0.184	0.038	0.080
Population size	10.84	870	0.0010	0.132	0.040	0.166
Latitude	8.81	870	0.003	−0.010	0.003	0.132
Aquatic	4.14	870	0.042	−0.117	0.058	0.101
Trophic level	39.42	870	<0.0001	0.132	0.021	0.069
Body mass	12.46	870	0.0004	0.189	0.054	0.080

Note

The model had the statistics likelihood ratio statistic LR = 432.29, *df* = 9, *p* < 0.0001. LR goodness of fit = 184.00, *df* = 857, *p* > 1.000. *N* is sample size, and *p* is probability. Effect size is Pearson's product–moment correlation coefficient.

## 4 DISCUSSION

Large flocks exceeding 100,000 individuals occur in the presence of favorable levels of environmental factors that usually limit local population size. Some bird species have exceedingly large flocks accounting for huge total numbers of individuals such as the extinct passenger pigeon *Ectopistes migratorius* (several billion individuals), brambling *Fringilla montifringilla* (418 million), red‐billed quelea *Quelea quelea* (217 million), and European starling (133 million). The passenger pigeon has been considered to represent a species apart due to its exceedingly large fluctuations in numbers and the crucial role of sociality for the maintenance of viability (Hung et al., 2014; Murray et al., 2017). We doubt that any other species resembles the passenger pigeon in its role of sociality for the maintenance of huge flocks. We found a highly uneven distribution of large flocks as shown by the pie diagram in Figure 2. We also found large spatial heterogeneity in distribution of large flocks. While China with the size of a continent only had one single observation, Europe had no <882 large flocks. Many similar examples of heterogeneous distribution suggest that resource availability must likewise be highly heterogeneous.

A number of correlates predict local population size as reflected by the size of large flocks. These factors include (a) predictors of productivity and (b) perturbations caused by humans such as time since the first records started, breeding season and trophic level. This first category includes factors such as total nitrogen use per km^2^, area used for agriculture, habitat degradation due to human activity and latitude that are all likely to directly or indirectly predict productivity. Colony size of reproducing animals will be limited because foraging distances will set an upper limit to the number of individuals that can breed in any given location (Ashmole, 1963; Furness & Birkhead, 1984; Jovani et al., 2015). This will mainly be a consequence of intraspecific competition, although effects of interspecific competition will likewise limit the number of individuals. Productivity will increase flock size as suggested by the present study showing effects of fertilizer, land‐use for agriculture, latitude, and trophic level on size of large flocks.

The second category of factors limiting the size of flocks is the effects of humans including perturbations and persecution. The passenger pigeon represents a prime example by being the most abundant species ever recorded until it went extinct. Schorger (1973) estimated that several billion individual passenger pigeons were present as late as around 1800. Such effects of humans may also include the temporal trend in flock size. The slope of the relationship between flock size for all birds and year with species as a random effect was −0.0033 (*SE* = 0.0011) for all years since 1637, which is the first year with information on flock size. Here, we have documented eight species of birds with significant decreases in flock size over time, but only a single species showing a positive relationship between flock size and year, and this ratio one to eight deviates from random expectation. There was a clear negative trend in slope of the relationship between flock size and year at least until the year 2000. This was even the case independent of elimination of all observations of passenger pigeons from the analyses (slope after elimination of all observations of passenger pigeons: estimate; *SE* = −0.0032 [0.0007]). Therefore, there was a clear and robust temporal trend in flock size covering the period until 2000. However, it is possible that other factors than human perturbation may have contributed to this slope because many variables may contribute to temporal trends.

A number of species are treated as pests, and considerable efforts have been made to eradicate these species. They include red‐billed quelea (Bruggers & Elliott, 1990) and starling (Feare & Craig, 1999). The red‐billed quelea numbered 217 million based only on the flocks included here, while the starling numbered 133 million. The red‐billed quelea is a pest of grain in Africa, while the starling can only be considered a secondary pest due to its consumption of cherries, olives, and other fruit.

Why do some species have large flocks, while others are rare and highly asocial? The evolution of sociality is a significant component of the major evolutionary transitions, and such transitions in sociality can be seen in huge aggregations of individuals in nests of termites, ants, and bees associated with relatedness and evolution of eusociality (Maynard Smith & Szathmáry, 1995). These cases differ from what we have described here because the huge flocks that we report are generally unrelated individuals. Indeed, the frequency of huge aggregations of individuals in eusocial insects is exceedingly rare compared to the more than 1,500 cases of large flocks in birds that sometimes reach as many as the several billion individuals in the now extinct passenger pigeon and the single flock of 61 million bramblings recorded in Switzerland in 1951–1953 (Géroudet, 1952). Here, we have focused on factors that are associated with resource limitation, but also factors that reduce access to resources such as aquatic compared to terrestrial habitats, seasonality that reduces access to resources to a few months of peak food availability during the main growing season, habitat degradation and aerial foraging that are linked to superabundant aggregated food.

It is obvious that this study suffers from a skewed distribution of amateur ornithologists in different parts of the world. In Western Europe and North America, the number of ornithologists is far larger than in South America, Africa, and Asia combined. In addition, online reporting systems have become widespread during the last couple of decades in Europe and North America providing huge numbers of observations of large flocks of birds.

This study has a number of perspectives. While it was entirely descriptive, we emphasize that several of the ideas presented here may be tested experimentally. For example, a reduction in fertilizer use estimated as the total amount of nitrogen released per km^2^ should result in a reduction in the number of large flocks. In addition, changes in threat status as affected by protection should result in an increase in flock size. Finally, change in fertilizer use should differentially affect the number of large flocks at the lowest trophic levels.

In conclusion, we have shown that the size of large flocks of birds can be considered to represent ecological indicators of a number of different environmental conditions including the level of total nitrogen fertilizers, threat status of bird species, and several others.

## 5 CONCLUSIONS

Large flocks of birds exceeding 100,000 individuals in size reveal large concentrations of resources and hence may constitute reliable indicators of resource availability. The magnitude of use of fertilizer per unit area and land‐use for agriculture may reflect large concentrations of resource availability. The size of large flocks changed with a number of additional indicators of resource availability such as habitat degradation by humans, trophic level, and latitude. A strong temporal decline in size of large flocks revealed a long‐term decline in the size of large flocks, and hence, changes in nutrients, reductions in nutrient cycling, and changes in flock size linked to trophic level. These findings suggest that the distribution and abundance of large flocks provide important information about the status of the environment.

## CONFLICT OF INTEREST

None declared.

## AUTHOR CONTRIBUTIONS

APM conceived and designed the experiments. APM and KL performed the study. APM analyzed the data. APM and KL wrote and edited the manuscript.

## ETHICAL APPROVAL

No approval was required for performing these entire observational studies.

## Data Availability

The data are available at Dryad (https://doi.org/10.5061/dryad.q0p0t3n).
