# Supplementary material for: The ecological significance of extremely large flocks of birds
Source: Ecol Evol. 2019 May 6;9(11):6559–67. doi: 10.1002/ece3.5234 (PMC6580271; doi:10.1002/ece3.5234)
Supplement: Supplementary file 1 [file ECE3-9-6559-s001.docx]

**Table S1.** Mean, range, SE and sample size for the variables included in this study.

| Variable | Mean | Range | Range | SE | Sample size |
| --- | --- | --- | --- | --- | --- |
| Number of individuals | 17,593,238 | 100,000 | 20,000,000 | 13,057,745 | 1564 |
| Year | 2000 | 1637 | 2019 | 0.62 | 1564 |
| Latitude | 40.03 | -78 | 82 | 0.75 | 1564 |
| Wing area (m^2^) | 0.030 | 0.012 | 0.129 | 0.0008 | 812 |
| Aspect ratio | 6.51 | 5.20 | 10.70 | 0.05 | 816 |
| Minimum population size | 2102,58 | 0.05 | 200,000 | 635.64 | 890 |
| Range | 49.06 | 0.001 | 466 | 1.64 | 1553 |
| Continuous threat status | -0.18 | -6 | 0 | 0.02 | 1564 |
| Human impact | 0.58 | 0 | 1 | 0.01 | 1564 |
| Body mass (g) | 748.10 | 13 | 12,000 | 42.66 | 1564 |
| Area (km^2^ x 10,000) | 6.64 1011 | 811 | 9.8 1013 | 2.09 1011 | 1481 |
| Mean total N (t) | 3,606,077 | 2636 | 1.72 107 | 128,003.37 | 1457 |
| Area agriculture (km^2^) | 2.96 1013 | 34,062 | 4.40 1015 | 9.32 1012 | 1481 |
| Aerial insectivore | 0.13 | 0 | 1 | 0.008 | 1564 |
| Aquatic habitat | 0.44 | 0 | 1 | 0.013 | 1564 |
| Trophic level | 1.93 | 1 | 3 | 0.018 | 1564 |
